# Scientific Content Analysis (SCAN) Cannot Distinguish Between Truthful and Fabricated Accounts of a Negative Event

**DOI:** 10.3389/fpsyg.2016.00243

**Published:** 2016-02-25

**Authors:** Glynis Bogaard, Ewout H. Meijer, Aldert Vrij, Harald Merckelbach

**Affiliations:** ^1^Clinical Psychological Science, Maastricht UniversityMaastricht, Netherlands; ^2^Psychology, University of PortsmouthPortsmouth, UK

**Keywords:** deception detection, scan, Scientific Content Analysis, Lie Detection, verbal cues, verbal credibility assessment

## Abstract

The Scientific Content Analysis (SCAN) is a verbal veracity assessment method that is currently used worldwide by investigative authorities. Yet, research investigating the accuracy of SCAN is scarce. The present study tested whether SCAN was able to accurately discriminate between true and fabricated statements. To this end, 117 participants were asked to write down one true and one fabricated statement about a recent negative event that happened in their lives. All statements were analyzed using 11 criteria derived from SCAN. Results indicated that SCAN was not able to correctly classify true and fabricated statements. Lacking empirical support, the application of SCAN in its current form should be discouraged.

## Introduction

Research has revealed that non-verbal cues (e.g., behavioral cues such as gaze aversion, sweating) are faint and differences between liars and truth tellers are small at best (DePaulo et al., 2003; Sporer and Schwandt, 2007). However, findings about verbal cues are less variable and are more strongly related to deception (Bond and DePaulo, 2006; Vrij, 2008b). Verbal cues (or content cues) are cues that can be found in the content and meaning of a statement, such as the number of details that are included in a story (e.g., he had a large spider tattoo in his neck). Indeed, lying has been shown to result in qualitative differences between deceptive and truthful language. As a result, various verbal credibility assessment tools have been developed that address these content criteria within statements. Although the exact content criteria included may differ depending on the method, the procedure is highly similar. The presence of the criteria within the statements is carefully checked, and based on the presence or absence of the various criteria, a conclusion is drawn about its truthfulness.

One example of such a content criterion is “quantity of details”. In order to fulfill this criterion, a statement has to be rich in details, such as mentioning places (e.g., it happened in the kitchen), times (e.g., on Sunday evening at 8 p.m.), descriptions of people and objects (e.g., a tall man with bright blue eyes), etc. Additionally, deceit has been related to the use of fewer personal pronouns (e.g., using “the house” instead of “our house”) and fewer negations (e.g., no, never, not), using less perceptual information (e.g., “I could smell the alcohol in his breath”), less details overall and shorter statements (Newman et al., 2003; Masip et al., 2005; Hauch et al., 2014; Amado et al., 2015). As mentioned previously, several methods have been developed to address these issues.

Two well-established credibility assessment tools that tap into such content differences are the Criteria Based Content Analysis (CBCA) and Reality Monitoring (RM). For CBCA, two theoretical assumptions have been presented by Köhnken (1996). First, lying is seen as more cognitively challenging that telling the truth. Second, liars are expected to be more concerned with impression management than truth tellers. More precisely, a first subset of CBCA criteria is included because they are deemed too difficult to fabricate (e.g., descriptions of interactions with the perpetrator). Hence, their presence in a statement indicates an actual experience. The remainder of the CBCA criteria are concerned with the way an interviewee presents his or her story. It is expected that liars are concerned with how they are viewed by others and therefore leave out information that can possibly damage their view of being an honest person (e.g., mentioning self-deprecating information). Consequently, a truthful person is more likely to include these criteria in their statement than a deceptive person. RM, in contrast, is derived from memory research and holds that memories of real events are obtained through sensory processes, making them more clear, sharp, and vivid. Fabricated statements, on the other hand, are the result of fantasy and are usually more vague and less concrete (Johnson and Raye, 1981). Indeed, various studies reported supportive evidence for these methods. Their overall accuracy for detecting deceit varies around 70%, and is considerably higher than chance level (Undeutsch, 1967; Johnson and Raye, 1981; Steller and Köhnken, 1989; Masip et al., 2005; Vrij, 2005; Amado et al., 2015).

Despite the research showing above chance accuracy for CBCA and RM, their field use seems limited. A third method – that is used by Law enforcement worldwide – is Scientific Content Analysis (SCAN). SCAN was developed by former Israeli polygraph examiner Avinoam Sapir (2005), who – based on his experience with polygraph examinees – argues that people who tell the truth differ from liars in the type of language they use. Based on these assumed differences, Sapir developed criteria that, according to him, can assist in differentiating between true and fabricated statements, but without reporting a theoretical foundation as to why these specific criteria should differ. For example, SCAN includes the criterion “social introduction”. It is argued that people who are described in the statement should be introduced with name and role (e.g., My friend, John). If a person leaves out information (e.g., We stole the key), so leaving out the name, role or both, this indicates deception. Another criterion is the “structure of the statement”. According to SCAN, 20% of the statement should consist of information that led up to the event, 50% should be about the main event and 30% of the statement should be about what happened after the event. The more the statement deviates from this structure, the higher the likelihood that the statement is deceptive. Yet, in contrast to CBCA and RM, no theoretical rationale is presented, and there is no evidence that these criteria are actually diagnostic (Nahari et al., 2012; Bogaard et al., 2014a; Vanderhallen et al., 2015).

Research about SCAN is scarce, although the method is used worldwide (e.g., Australia, Belgium, Canada, Israel, Mexico, UK, US, the Netherlands, Qatar, Singapore, South Africa) and is also used by federal agencies, military law enforcement, private corporations, and social services (retrieved from www.lsiscan.com/id29.htm). Moreover, the third author asked during an investigative interviewing seminar which lie detection tool was used by the practitioners in the audience. These practitioners came from many different countries and the most frequent answer was SCAN (Vrij, 2008a). In a typical SCAN procedure, the examinee is asked to write down “everything that happened” in a particular period of time, to get a “pure version” of the facts (Sapir, 2005). This pure version is typically obtained without the interviewer interrupting or influencing the examinee. Next, a SCAN trained analyst investigates a copy of the handwritten statement, using several criteria that are described throughout the SCAN manual (Sapir, 2005). Criteria that are present within the written statements are highlighted according to a specific color scheme, circled or underlined. The presence of a specific criterion can either indicate truthfulness or deception, depending on the criterion itself. This SCAN analysis is then used to generate questions that could elucidate important details within the statement, and/or to make a judgment of the veracity of the statement. Although SCAN is used worldwide, it lacks a well-defined list of criteria, as well as a standardized scoring system. Bogaard et al. (2014b) has shown that 12 criteria primarily drove SCAN in sexual abuse cases, largely overlapping with the criteria list described in Vrij (2008a). Only six published studies examined the validity of SCAN (Driscoll, 1994; Porter and Yuille, 1996; Smith, 2001; Nahari et al., 2012; Bogaard et al., 2014a; Vanderhallen et al., 2015) of which only four were published in peer reviewed journals. The two studies that were not published in peer reviewed journals [Driscoll (1994) and Smith (2001)] were both field studies investigating suspect statements.

Driscoll (1994) investigated 30 statements that were classified as either apparently accurate or doubtful. With the help of SCAN, 84% of the statements could be classified correctly. In the study of Smith, five groups of experts were asked to analyze 27 statements. These statements were previously classified by police officers as truthful, false, or undecided. This classification was made on the basis of confessions and supportive evidence. Three groups consisted of SCAN trained officers that had minimal, moderate, or extensive experience with using SCAN. The two other groups consisted of newly recruited officers and experienced officers. The first three groups used SCAN to analyze the statements, while the latter two groups judged the veracity of the statements without using SCAN. Overall, the SCAN groups correctly judged 78% of the statements, which was similar to the accuracy of the experienced officers. At first glance, these results seem to support SCAN. Yet, in both studies ground truth of the statements was unknown and statements were categorized as either truthful or doubtful without having hard evidence supporting this categorisation. Moreover, it cannot be excluded that the SCAN outcome influenced the course of the investigation, and therefore the confessions and supporting evidence that was gathered. A typical problem that can occur in such studies is that errors are systematically excluded from the sample. For example, if a statement is erroneously judged as truthful, no further investigation takes place. This means that no evidence will be found revealing that an error has been made, and such erroneous classifications are then excluded from the sample. This way of selecting the sample may therefore be biased to overestimate SCAN’s accuracy (for more information see Iacono, 1991; Meijer et al., 2016). Moreover, in Smith’s study, it was unclear whether the three undecided statements were included in the reported analyses (Armistead, 2011).

The following four studies investigating SCAN were published in peer-reviewed journals. Porter and Yuille (1996) resolved the problem of ground truth by asking participants to commit a mock crime. However, they only investigated three SCAN criteria (i.e., unnecessary connectors, use of pronouns, and structure of the statement), and results indicated no significant differences between true and fabricated statements concerning these criteria. Nahari et al. (2012) asked six independent raters to assess the presence of 13 SCAN criteria within various true and fabricated statements. Results showed that SCAN did not discriminate between truthful and fabricated statements, a conclusion that was also supported by Bogaard et al. (2014a). In their study, participants were asked to write down one truthful and one fabricated autobiographical statement about a negative event that recently happened to them. Two raters indicated the presence of 12 SCAN criteria, but no significant differences emerged between truth tellers and liars. Vanderhallen et al. (2015), finally, asked SCAN trained police officers to classify four statements as either truthful or deceptive based on SCAN, and compared their accuracy to students and police officers who made this classification without the help of SCAN. The SCAN group had an average accuracy of 68%, police officers without SCAN 72%, and students 65%. The accuracy of the SCAN group did not significantly differ from the police officers who did not use SCAN. Consequently, from these results it was concluded that SCAN did not have an incremental value in detecting deceit.

Given that SCAN is used worldwide in police investigations, providing support, or the lack thereof, is not trivial (Meijer et al., 2009). Using a data set of 234 statements, the current study aimed at extending previous SCAN findings, and to investigate whether the different SCAN criteria can actually discriminate between truthful and fabricated statements. Although Nahari et al. (2012), Bogaard et al. (2014a), and Vanderhallen et al. (2015) investigated SCAN, they mainly focused on the SCAN total scores, and not on the separate criteria, or the accuracy of SCAN. Separate criteria scores were reported, but their power was too low to make any conclusions from these results. In contrast, Nahari et al. (2012) asked participants to perform a mock crime, meaning that the statements that were analyzed with SCAN were restricted to “false denials” (i.e., people who performed the mock crime but lied about it). Moreover, in the study of Vanderhallen et al. (2015) four statements on traffic accidents were used. The statements included in our study are broader than false denials or traffic accidents, as we requested participants to write about a negative autobiographical event. In this way, participants not only reported false denials, but also false allegations (i.e., stating they fell victim to a crime, while in fact they were not). Participants could report about whatever they preferred, thereby including various topics, as would also be the case in police investigations where SCAN is usually applied.

## Materials and Methods

### Ethics Statement

The study was approved by the standing ethical committee of the Faculty of Psychology and Neuroscience, Maastricht University.

### Participants

All participants (*N* = 117) were first and second year health sciences students (i.e., Mental health or Psychology) of Maastricht University (37 men). The data of 85 participants were collected specifically for this study, while the remaining 32 came from the control group of Bogaard et al. (2014a). Instructions for these two datasets were identical, and they were combined to increase power. We report the analysis for the entire sample below, but also include the findings for the new dataset in Appendix B.

Participants could choose whether they wanted to receive one course credit or a 7,5 €; gift voucher for their participation. Approximately 50 students chose the gift voucher over the course credit. All participants read and signed a letter of Informed Consent before they took part in this study. Participants had a mean age of 21 years (*SD* = 2.35). The experiment was approved by the appropriate standing ethical committee.

### Procedure

Upon arrival in the lab, participants were told that the study was about the accuracy of verbal lie detection methods. Participants were asked to write about a truthful and a fabricated event. The order in which participants wrote these statements was randomized. Approximately half of the participants started with the truthful statement, the other half started with the fabricated statement. For the truthful statement participants received the following instruction: “For this study we ask you to think about an event you actually experienced. More specifically, this event should be about a recent negative experience; think about a financial, emotional or physical negative event you’ve been through the past months.” For the fabricated statement participants received the following instruction: “For this study we ask you to think about an event that you have not actually experienced. This event should be about a recent negative experience; think about a financial, emotional, or physical negative event you could have been through the past months. This event should not be based on something that actually happened to you or your friends or family. Please pretend as if this event took place somewhere in the previous months. Although the story should be fabricated, the statement should consist of a realistic scenario.” After the instruction, participants had the opportunity to think about a real and a fabricated story for a maximum of 5 min. Participants were assured that their stories would be treated confidentially and anonymously. They were told that the length of the stories should be approximately one written page (A4). No time limit was set for the production of the statements.

### Statement Coding

After participants finished their stories, these were analyzed by four raters. One rater completed the three-day SCAN course. The other three raters received a 2-h training about SCAN, using the SCAN manual (Sapir, 2005), given by the SCAN trained rater. Moreover, they received the appropriate pages of Vrij (2008a) about SCAN (Chapter 10; 282-287). During the training all 12 criteria were discussed separately and examples of the specific criteria were presented and discussed. Next, raters received two practice statements of one page each, and were asked to analyze these statements. After all raters analyzed these statements, their analyses were discussed and questions they still had about SCAN were answered. When the training was completed, raters started analyzing the statements.

Although the raters were not blind to the aim of the study, they were blind to the veracity of the statements. The first author served as one of the raters, the other raters were not otherwise involved in the study and were research assistants of the first author. The rater who completed the original SCAN training scored all 234 statements, while the other three raters scored approximately 80 statements each. In order to control for potential order effects, the sequence of the statements to be scored was varied from rater to rater. Rater A scored all statements in the order of 1–234, while the other raters scored the statements in the reverse order (rater B started from 79 to 1, rater C started from 157 to 80 and rater D from 234 to 158).

A total of 12 criteria (Vrij, 2008a) were coded within the statements. According to SCAN, seven of these criteria indicate truthfulness: (1) denial of allegations, (2) Social introductions, (3) Structure of the statement, (4) Emotions, (5) Objective and subjective time, (6) First person singular, past tense, (7) Pronouns, while the remaining five indicate deception: (8) Change in language (9) Spontaneous corrections (10) Lack of conviction or memory (11) Out of sequence and extraneous, (12) Missing information. See Appendix A for a complete description of the different criteria. All criteria that are expected to indicate truthfulness were scored on a three-point scale ranging from 0 (not present) to 2 (strongly present), while the five criteria that are expected to indicate deception were scored in reverse, ranging from -2 (strongly present) to 0 (not present). By using this scoring system, a higher score indicates a higher likelihood that the statement is truthful and vice versa.

## Results

### Inter-Rater Reliablility

Inter-rater reliability was calculated by means of Cohen’s Kappa for each of the 12 separate criteria. The Kappa values for the truthful statements varied from 0.60 to 1 with an average Cohen’s Kappa of 0.77. The Kappa values for the fabricated statements varied from 0.65 to 1, with an average kappa of 0.78. These results indicated that there is high agreement between the raters (Landis and Koch, 1977). Because variance was low for several criteria, Cohen’s Kappa could give a distorted image of the actual inter-rater reliability. Therefore, we also included inter-rater agreement calculated by means of percentage agreement and its presence in the statement. Therefore, we dichotomized the original data set with presence coded as 1 and absence as 0. High agreement was achieved for all SCAN criteria ranging from 80.34 to 100% with an average of 90.56%. The scoring of the three raters was always compared to those of the rater that completed the SCAN training. As reliability showed to be sufficient, this also showed that our 2-h SCAN training was sufficient to score the investigated SCAN criteria reliably.

### Data Analysis

Because the inter-rater reliability was high, we averaged the scores of the two raters for each criterion. Due to the nature of our instructions (i.e., autobiographical statements) the first criteria could not have been coded in the statements. As such, we have left out “denial of allegations” in the following analysis. Next, we calculated the sum scores for each statement by summing up the averaged scores of the separate criteria. To investigate the discriminability of SCAN, we conducted several Generalized Estimation Equation (GEE) analyses (see for example Burton et al., 1998); one for each separate criterion. Moreover, we conducted a paired samples *t*-test for the sum score, and a discriminant analysis to test SCAN’s predictive power concerning the veracity of the statements.

### Number of Words

The length of the statements did not significantly differ between the true (*M* = 265.42; *SD* = 85.48) and fabricated statements (*M* = 261.86; *SD* = 88.12) [*t*(116) = 0.63, *p* = 0.53, *d* = 0.04].

### SCAN Criteria Scores

**Table 1** shows the mean differences in each of the SCAN criteria as a function of veracity. To analyze the separate criteria, we have dichotomized our data by recoding presence as 1 (regardless of whether the score was a 1 or a 2) and absence as 0. Next, we analyzed the data with GEE in order to investigate the differences between truthful and fabricated statement for each of the separate criteria. Due to very low variability of the criterion “pronouns” (i.e., it was present in almost all of the statements), this criterion was left out of the analysis. To correct for multiple testing we used an alpha level of 0.01. As **Table 2** shows, only one criterion significantly differed between the statements, namely “Change in language”. Participants included more changes in language in their fabricated statements compared to their truthful statements. This criterion was present in 29 out of 117 fabricated statements (24.8%) and in 14 out of 117 true statements (12%). In Appendix B (Table B1) we have presented the results of only the new data, and results showed again that “Change in language” significantly differed between statements.

**Table 1:** Means, standard deviations and percentage present for each SCAN criterion as a function of veracity.

SCAN criteria	True			Fabricated		
	Mean	*SD*	% present	Mean	*SD*	% present
1. Denial of allegations	0	0	0	0	0	0
2. Social introduction	1.26	0.81	76.90	1.40	0.71	87.20
3. Structure of the statement	0.73	0.60	67.50	0.59	0.60	56.40
4. Emotions	1.05	0.62	83.80	0.95	0.65	76.10
5. Objective and Subjective time	0.71	0.65	62.40	0.79	0.65	69.20
6. First pers sing, past tense	1.59	0.63	92.30	1.60	0.60	94.00
7. Pronouns	1.68	0.49	97.40	1.69	0.50	97.40
8. Change in language	-0.09	0.27	12.00	-0.23	0.43	24.80
9. Spontaneous corrections	-0.61	0.62	56.40	-0.64	0.63	58.10
10. Lack of conviction or memory	-0.16	0.36	18.80	-0.14	0.33	16.40
11. Out of sequence and extraneous info	-0.18	0.38	21.40	-0.20	0.43	22.20
12. Missing information	-0.64	0.55	75.00	-0.67	0.52	67.50

**Table 2:** Overview of parameters from the GEE analysis.

Criteria	Beta estimate	*SE*	95% CI	Odds ratio
8. Change in language	-0.89	0.36	-1.59, -0.18	**0.79**
2. Social introduction	-0.713	0.34	-1.37, -0.05	0.51
4. Emotions	0.48	0.27	-0.06, 1.03	0.23
3. Structure of statement	0.47	0.26	-0.04, 0.99	0.22
5. Objective and subjective time	-0.31	0.21	-0.73, 0.12	0.10
6. First pers sing, past tense	-0.27	0.51	-1.26, 0.72	0.07
10. Lack of conviction or memory	0.24	0.31	-0.36, 0.85	0.06
12. Missing information	-0.12	0.21	-0.53, 0.30	0.01
9. Spontaneous corrections	-0.07	0.22	-0.50, 0.36	0.00
11. Out of sequence and extraneous information	-0.05	0.28	-0.60, 0.50	0.00

*Significant difference between statement types, p = 0.01 is in bold.*

### SCAN Sum Scores

Results indicated that there were no differences in SCAN sum scores between true (*M* = 5.33; *SD* = 2.10) and fabricated (*M* = 5.15; *SD* = 2.25) statements [*t*(116) = 0.77, *p* = 0.44, *d* = 0.12].

Lastly, we conducted a discriminant analysis to investigate whether the SCAN criteria were able to predict veracity. As can be seen in **Table 3**, only one significant mean difference was observed, and this was for “Change in language” (*p* < 0.01). The discriminate function revealed a low association between veracity and SCAN criteria, only accounting for 7.20% of the variability. Closer analysis of the structure matrix revealed that three criteria that had moderate discriminant loadings (i.e., Pearson coefficients), these were – again – “Change in language” (0.664), “Structure of the statement” (0.412), and “Social introduction” (-0.353). The uncorrected model resulted in correct classification of 59% of the truth tellers, and 65% of the liars. The cross-validated classification, however, showed that 49.60% of the liars and 53% of the truth tellers were correctly classified, thereby showing that SCAN performed around chance level. In Appendix B (Table B2), we have presented the results of only the new data, and results showed to be similar. The uncorrected model resulted in a correct classification of 63% of the truth tellers, and 58% of the liars. The cross-validated classification showed that 50% of the liars and 55% of the truth tellers were correctly classified, again showing that SCAN performed around chance level.

**Table 3:** Detailed overview of discriminant analysis coefficients.

Criteria	Mean	*SD*	Structure matrix	Discriminant function coefficients
8. Change in language	-0.16	0.37	0.66	1.82
3. Structure of statement	0.66	0.60	0.41	0.79
4. Emotions	1.00	0.63	0.29	0.67
9. Spontaneous corrections	-0.62	0.62	0.07	0.23
11. Out of sequence and extraneous information	-0.19	0.40	0.10	0.20
12. Missing information	-0.65	0.53	0.12	0.12
7. Pronouns	1.69	0.49	-0.03	-0.09
6. First pers sing. past tense	1.59	0.61	-0.04	-0.14
10. Lack of conviction or memory	-0.15	0.35	-0.11	-0.21
5. Objective and subjective time	0.75	0.65	-0.23	-0.43
2. Social introduction	1.33	0.77	-0.35	-0.53

## Discussion

In the current study, we failed to find support for SCAN as a lie detection method. The total SCAN score did not significantly differ between true and fabricated statements, so confirming previous results (Nahari et al., 2012; Bogaard et al., 2014a). Interestingly, for a subset of our data CBCA and RM sum scores were coded and did discriminate between the truthful and fabricated statements (Bogaard et al., 2014a). As such, it seems that the absence of significant SCAN findings cannot be attributed to the quality of the statements used in this study. Furthermore, we investigated the separate SCAN criteria, and only one criterion “Change in language” significantly differentiated between true and fabricated statements; participants changed their language more in their fabricated statements compared to their truthful statements.

Interestingly, the criterion “Change in language” is not described in other verbal credibility methods (e.g., CBCA, RM). Therefore, our findings concerning this criterion are noteworthy. Sapir (2005) explained in his manual that especially words describing family members (e.g., mother, father, dad, mom, etc.), people (e.g., someone, individual, man, guy, etc.), communication (e.g., told, spoke, talked, etc.), transport (e.g., vehicle, car, truck, etc.) and weapons (e.g., gun, rifle, revolver, pistol, etc.) should be investigated carefully. The idea is that such a change indicates something has altered in the mind of the writer. When the events in the statements justify this change it does not indicate deception *per se*, however, in all other cases these changes indicate deceit. But what exactly is meant by a justification is not described in the manual. Consequently, due to the absence of clear guidelines on verifying whether a change is justified, the current study scored all changes in language as a cue to deceit, and might therefore differ from how SCAN is used in practice.

Both the analyses of the SCAN sum score and the discriminant analysis showed SCAN did not perform above chance level. This chance level performance can be understood when looking at various contradicting interpretations of its criteria compared with CBCA. More precisely, both methods describe “spontaneous corrections” and “lack of conviction or memory”, but differ in their use. For CBCA both criteria are interpreted as a sign of truthfulness, while for SCAN both criteria are interpreted as a sign of deceit. Commonsensically, only one interpretation can be correct. As CBCA is far more embedded in the scientific literature and has been shown to detect deceit above chance level (Vrij, 2005; Amado et al., 2015), CBCA’s interpretations should be favored over SCAN. Also, SCAN does not consider criteria involved in judging distinctive types of details. Both CBCA and RM consist of various types of details that have to be checked. For example, with these methods it is checked whether there is information in the statement about when (i.e., temporal details) and where (i.e., spatial details) the event took place, about what the writer saw during the event (i.e., visual details) and whether there were any other perceptual details (i.e., smells, tastes, sensations, sounds). Research showed that especially these types of criteria are significantly more present in truthful compared to fabricated statements (DePaulo et al., 2003; Masip et al., 2005; Vrij, 2005).

Relatedly, recent meta-analytical research reveals that passively observing cues only has a limited influence on our deception detection abilities, as most of these cues are generally weak (Hartwig and Bond, 2011). The authors argue we should actively increase the verbal and non-verbal differences between liars and truth tellers. Various techniques have already been suggested, such as focusing on unanticipated questions during the interrogation (Vrij et al., 2009), applying the Strategic Use of Evidence technique (Granhag et al., 2007) or inducing cognitive load (Vrij et al., 2006, 2008, 2011, 2012). SCAN fails to actively influence the information that is provided by the interviewee, which potentially contributes to its chance performance.

Finally, users of SCAN may argue that the way SCAN is tested in laboratory studies such as these, is far from how it is applied in the field, and that the results therefore do not translate. However, the diagnostic value of SCAN and its criteria lies within its capabilities of discriminating between truthful and fabricated statements. SCAN makes no assumptions as to *why* or *when* these differences between truths and lies occur, only that they occur. As such, also laboratory studies – for example where participants are asked to fabricate a negative event – should be able to pick up such differences, if they exist. Moreover, it has proven to be exceptionally difficult to test the accuracy of SCAN in field studies as the reliability of SCAN has shown to be extremely low (Bogaard et al., 2014b; Vanderhallen et al., 2015). The only way to control for this low reliability is to use a more standardized scoring system, as we have done so in the current study. For example, as is mentioned previously, SCAN does not consist of a fixed list of criteria, and the criteria are not scored on a scale. In field studies, SCAN analysts write a report about the presence or absence of the criteria, and on the basis of this report, they make a conclusion about the truthfulness of the statement. As such, it is unclear how many criteria are actually taken into consideration when making a judgment, and whether these criteria are weighed equally.

## Conclusion

Scientific Content Analysis has no empirical support to date, and fails to include criteria investigating different types of details. Only one criterion showed potential for lie detection research, but has to be investigated more thoroughly in order to overcome the problems that are inherent to SCAN and its criteria (e.g., vague description, ambiguous interpretation). As a result, we discourage the application of SCAN in its current form.

## Author Contributions

All authors have made substantial contributions to the conception and design of the work, GB acquired and analyzed the data, and all authors interpreted the data and revised the article concerning its content and approved the current version.

## Conflict of Interest Statement

The authors declare that the research was conducted in the absence of any commercial or financial relationships that could be construed as a potential conflict of interest.

## References

[B1] Amado B. G. Arce R. Fariña F. (2015). Undeutsch hypothesis and criteria based content analysis: a meta-analytic review. *Eur. J. Psychol. Appl. Legal Context* 7 1–10. 10.1016/j.ejpal.2014.11.002

[B2] Armistead T. W. (2011). Detecting deception in written statements: the british home office study of scientific content analysis (Scan). *Policing* 34 588–605. 10.1108/13639511111180225

[B3] Bogaard G. Meijer E. Vrij A. (2014a). Using an example statement increases information but does not increase accuracy of Cbca, Rm, and Scan. *J. Investig. Psychol. Offender Profil.* 11 151–163. 10.1002/jip.1409

[B4] Bogaard G. Meijer E. Vrij A. Broers N. J. Merckelbach H. (2014b). Scan Is Largely driven by 12 criteria: results from field data. *Psychol. Crime Law* 20 430–449. 10.1080/1068316X.2013.793338

[B5] Bond C. F. DePaulo B. M. (2006). Accuracy of deception judgments. *Pers. Individ. Differ.* 10 214–234. 10.1207/s15327957pspr1003_2 16859438

[B6] Burton P. Gurrin L. Sly P. (1998). Tutorial in biostatistics. Extending the Simple linear regression model to account for correlated responses: an introduction to generalized estimating equations and multi-level mixed modeling. *Stat. Med.* 17 1261–1291. 10.1002/(SICI)1097-0258(19980615)17:11<1261::AID-SIM846>3.0.CO;2-Z 9670414

[B7] DePaulo B. M. Lindsay J. J. Malone B. E. Muhlenbruck L. Charlton K. Cooper H. (2003). Cues to deception. *Psychol. Bull.* 129 74–118. 10.1037/0033-2909.129.1.74 12555795

[B8] Driscoll L. (1994). A validity assessment of written statements from suspects in criminal investigations using the scan technique. *Police Stud.* 4 77–88.

[B9] Granhag P. A. Strömwall L. A. Hartwig M. (2007). The sue-technique: the way to interview to detect deception. *Forensic Update* 88 25–29.

[B10] Hartwig M. Bond C. F. (2011). Why do lie-catchers fail? A lens model meta-analysis of human lie judgments. *Psychol. Bull.* 137 643–659. 10.1037/a0023589 21707129

[B11] Hauch V. Blandón-Gitlin I. Masip J. Sporer S. L. (2014). Are computers effective lie detectors? A meta-analysis of linguistic cues to deception. *Pers. Soc. Psychol. Rev.* 19 307–342. 10.1177/1088868314556539 25387767

[B12] Iacono W. G. (1991). “Can we determine the accuracy of polygraph tests?,” in *Advances in Psychophysiology*, eds Jennings J. R. Ackles P. K. Coles M. G. H. (London: Jessica Kingsley Publishers), 201–207.

[B13] Johnson M. K. Raye C. L. (1981). Reality monitoring. *Psychol. Rev.* 88 67–85. 10.1037/0033-295X.88.1.67

[B14] Köhnken G. (1996). “Social psychology and the law,” in *Applied Social Psychology*, eds Semin G. Fiedler K. (London: Sage Publication), 257–282.

[B15] Landis J. R. Koch G. G. (1977). The measurement of observer agreement for categorical data. *Biometrics* 33 159–174. 10.2307/2529310 843571

[B16] Masip J. Sporer A. L. Garido E. Herrero C. (2005). The detection of deception with the reality monitoring approach: a review of the empirical evidence. *Psychol. Crime Law* 11 99–122. 10.1080/10683160410001726356

[B17] Meijer E. H. Verschuere B. Gamer M. Merckelbach H. Ben-Shakhar G. (2016). Deception detection with behavioral, autonomic, and neural measures: conceptual and methodological considerations that warrant modesty. *Psychophysiology* 10.1111/psyp.12609 [Epub ahead of print]. 26787599

[B18] Meijer E. H. Verschuere B. Vrij A. Merckelbach H. Smulders F. Leal S. (2009). A call for evidence-based security tools. *Open Access J. Forensic Psychol.* 1 1–4.

[B19] Nahari G. Vrij A. Fisher R. P. (2012). Does the truth come out in the writing? Scan as a lie detection tool. *Law Hum. Behav.* 36 68–76. 10.1037/h0093965 22471387

[B20] Newman M. L. Pennebaker J. W. Berry D. S. Richards J. M. (2003). Lying words: predicting deception from linguistics styles. *Pers. Soc. Psychol. Bull.* 29 665–675. 10.1177/0146167203029005010 15272998

[B21] Porter S. Yuille Y. C. (1996). The language of deceit: an investigation of the verbal clues to deception in the interrogation context. *Law Hum. Behav.* 20 443–458. 10.1007/BF01498980

[B22] Sapir A. (2005). *The Lsi Course on Scientific Content Analysis (Scan)*. Phoenix, AZ: Laboratory for Scientific Interrogation.

[B23] Smith N. (2001). Reading between the lines: an evaluation of the scientific content analysis technique (Scan). *Police Res. Series Paper* 135 1–42.

[B24] Sporer S. L. Schwandt B. (2007). Moderators of nonverbal indicators of deception. *Psychol. Public Policy Law* 13 1–34. 10.1037/1076-8971.13.1.1

[B25] Steller M. Köhnken G. (1989). “Criteria based statement analysis,” in *Psychological Methods in Criminal Investigation and Evidence*, ed. Raskin D. C. (New York, NY: Springer), 217–245.

[B26] Undeutsch U. (1967). “Beurteilung der glaubhaftigkeit von aussagen,” in *Handbuch Der Psychologie Vol 11: Forensische Psychologie*, ed. Undeutsch U. (Göttingen: Hogrefe).

[B27] Vanderhallen M. Jaspaert E. Vervaeke G. (2015). Scan as an investigative tool. *Police Pract. Res.* 1–15. 10.1080/15614263.2015.1008479

[B28] Vrij A. (2005). Criteria based content analysis: a qualitative review of the first 37 studies. *Psychol. Public Policy Law* 11 3–41. 10.1037/1076-8971.11.1.3

[B29] Vrij A. (2008a). *Detecting Lies and Deceit: Pitfalls and Opportunities*. Chichester: Wiley.

[B30] Vrij A. (2008b). Nonverbal dominance versus verbal accuracy in lie detection: a plea to change police practive. *Crim. J. Behav.* 35 1323–1336. 10.1177/0093854808321530

[B31] Vrij A. Fisher R. Mann S. Leal S. (2006). Detecting deception by manipulating cognitive load. *Trends Cogn. Sci.* 10 141–142. 10.1016/j.tics.2006.02.003 16516533

[B32] Vrij A. Fisher R. Mann S. Leal S. (2008). A cognitive load approach to lie detection. *J. Investig. Psychol. Offender Profiling* 5 39–43. 10.1002/jip.82

[B33] Vrij A. Granhag P. A. Mann S. Leal S. (2011). Outsmarting the liars: toward a cognitive lie detection approach. *Curr. Dir. Psychol. Sci.* 20 28–32. 10.1177/0963721410391245

[B34] Vrij A. Leal S. Granhag P. A. Mann S. Fisher R. Hillman J. (2009). Outsmarting the liars: the benefit of asking unanticipated questions. *Law Hum. Behav.* 33 159–166. 10.1007/s10979-008-9143-y 18523881

[B35] Vrij A. Leal S. Mann S. Fisher R. (2012). Imposing cognitive load to elicit cues to deceit: inducing the reverse order technique naturally. *Psychol. Crime Law* 18 579–594. 10.1080/1068316X.2010.498422

