# Supplementary material for: Scientific Content Analysis (SCAN) Cannot Distinguish Between Truthful and Fabricated Accounts of a Negative Event
Source: Front Psychol. 2016 Feb 25;7:243. doi: 10.3389/fpsyg.2016.00243 (PMC4766305; doi:10.3389/fpsyg.2016.00243)
Supplement: Supplementary file 1 [file Data_Sheet_1.DOCX]

**Appendix A**

SCAN criteria (derived from Vrij (2008a)).

1. **Denial of allegations**: Refers to whether the examinee directly denies the allegation in the statement by stating “I did not…”. This criterion assumes that a truthful person is more likely to directly deny his or her involvement in the act.
2. **Social introduction**: Refers to how the persons described in the statement are introduced. People that are described within a statement should be introduced in an unambiguous way, usually by mentioning their name and role (e.g., My wife, Susan). Deviations from this type of introduction indicate deception.
3. **Structure of the statement**: Refers to the balance of the statement. In a truthful statement 20% is used to describe activities leading up to the event, the next 50% to describe the actual event, and the final 30% to discuss what happened after the event.
4. **Emotions**: Refers to where there are emotions described in the statement. Usually emotions should be described in the epilogue of the statement. When emotions are already included within the description of the prologue (before the actual event), this indicates deception. For example, “On Saturday something strange happened to me, I was really scared” (emotions before main event) or “when he was gone, I felt disgusted with myself” (emotions after the main event). The former would indicate deception, the latter truthfulness.
5. **Objective and subjective time**: Refers to how different time periods are covered in the statement. Objective time refers to the actual duration of events described, whereas subjective time refers to the number of words used describing these events. On average a writer is expected to need three or four lines per hour when describing one day. Large deviations from this pace suggest deception.
6. **First person singular, past tense**: Refers to the format in which a statement is written. This is called the test of commitment, and holds that a truthful person will write the statement in first person singular, past tense. Deviations from past tense or writing in the third person could indicate a lack of commitment and hence could indicate deception.
7. **Pronouns**: Refers to the use of pronouns in the statement. When pronouns (e.g., “he”, “mine”, “my”) are missing in the statement, or more pronouns are expected, this could suggest that the writer wants to distance him/herself from the statement. This indicates deception. For example, when a writer refers to his car as “the car” and never as “my car” this could mean he is being deceptive about what happened to the car.
8. **Change in language**: Refers to the change of terminology or vocabulary in the statement. This is especially important for words that are related to categories such as family members, people, communication, transport or weapons. When a change of language is obvious in a statement (e.g., knife to blade) but no justification is given for such a change, this indicates deception. A change in language indicates that something has altered in the mind of the writer.*
9. **Spontaneous corrections**: Refers to all corrections that are made within the statements. Before the writer starts with the statement s/he is instructed not to cross anything out, and when the writer fails to follow this instruction, this indicates deception.*
10. **Lack of conviction or memory**: Refers to when the writer is vague about certain elements within the statement (e.g. “I think…”, “I guess…”) or when the writer admits he or she has forgotten something (e.g., “I do not remember how we got to the house”). Lack of memory indicates deceit.*
11. **Out of sequence and extraneous information**: Examines whether the statement includes information that is given by the writer, but has no apparent meaning for the reader or whether there is strange or irrelevant information within the statement. Whether the information is seen as strange or irrelevant depends on the statement itself. It is thought that by including this type of information, the writer is distracting the reader to hide more important information. This is seen as a sign of deception.*
12. **Missing information**: Refers to phrases in the statement that indicate some information has been left out. For example, words such as “after a while”, “shortly thereafter”, or “the next thing I remember” all indicate there is information missing within the statement. This is especially relevant when the writer is discussing the main event. Missing information during the main event could indicate that the writer is deliberately hiding information, which indicates the person is deceptive.*

**Appendix** **B**

Table B1. *Overview of parameters from the GEE analysis of new data.*

| Criteria | Beta Estimate | SE | 95% CI | Odds ratio |
| --- | --- | --- | --- | --- |
| 8. Change in language | -1.08 | 0.44 | -1.94, -.23 | **1.18** |
| 2. Social introduction | -0.87 | 0.52 | -1.37, -0.05 | 0.75 |
| 4. Emotions | 0.73 | 0.43 | -0.12, 1.57 | 0.53 |
| 3. Structure of statement | 0.45 | 0.31 | -0.16, 1.07 | 0.21 |
| 5. Objective and subjective time | -0.46 | 0.28 | -1.01, 0.10 | 0.21 |
| 6. First pers sing. past tense | -0.31 | 0.54 | -1.38, 0.75 | 0.10 |
| 10. Lack of conviction or memory | 0.20 | 0.33 | -0.46, 0.86 | 0.04 |
| 12. Missing information | -0.21 | 0.33 | -0.87, 0.43 | 0.05 |
| 9. Spontaneous corrections | -0.21 | 0.29 | -0.78, 0.35 | 0.04 |
| 11. Out of sequence and extraneous information | -0.08 | 0.36 | -0.80, 0.63 | 0.01 |

*Note*. Significant difference between statement types, *p* = 0.01 is in bold.

Table B2. *Detailed overview of discriminant analysis coefficients derived from the new data.*

| Criteria | Mean | SD | Structure matrix | Discriminant function  coefficients |
| --- | --- | --- | --- | --- |
| 8. Change in language | -0.17 | 0.38 | 0.05 | 1.51 |
| 3. Structure of statement | 0.64 | 0.60 | 0.32 | 0.80 |
| 4. Emotions | 1.11 | 0.60 | -0.24 | 0.74 |
| 9. Spontaneous corrections | -0.71 | 0.63 | 0.36 | 0.34 |
| 11. Out of sequence and extraneous information | -0.16 | 0.41 | 0.14 | 0.48 |
| 12. Missing information | -0.80 | 0.50 | 0.12 | 0.23 |
| 7. Pronouns | 1.69 | 0.51 | -0.07 | 0.22 |
| 6. First pers sing. past tense | 1.59 | 0.63 | 0.10 | -0.42 |
| 10. Lack of conviction or memory | -0.20 | 0.39 | -0.34 | 0.13 |
| 5. Objective and subjective time | 0.91 | 0.66 | -0.19 | -0.66 |
| 2. Social introduction | 1.56 | 0.69 | 0.56 | -0.66 |
